# Hepatic Glucose Intolerance Precedes Hepatic Steatosis in the Male Aromatase Knockout (ArKO) Mouse

**DOI:** 10.1371/journal.pone.0087230

**Published:** 2014-02-10

**Authors:** Michelle L. Van Sinderen, Gregory R. Steinberg, Sebastian B. Jørgensen, Sarah Q. To, Kevin C. Knower, Colin D. Clyne, Jane Honeyman, Jenny D. Chow, Kerrie A. Herridge, Margaret E. E. Jones, Evan R. Simpson, Wah Chin Boon

**Affiliations:** 1 Prince Henry's Institute of Medical Research, Clayton, Victoria, Australia; 2 Florey Neuroscience Institutes, University of Melbourne, Parkville, Victoria, Australia; 3 Department of Anatomy and Developmental Biology, Monash University, Clayton, Victoria, Australia; 4 St Vincent's Institute of Medical Research and Department of Medicine, University of Melbourne, Fitzroy, Victoria, Australia; 5 Department of Medicine, Division of Endocrinology and Metabolism, McMaster University, Hamilton, Ontario, Canada; Wageningen University, Netherlands

## Abstract

Estrogens are known to play a role in modulating metabolic processes within the body. The Aromatase knockout (ArKO) mice have been shown to harbor factors of Metabolic syndrome with central adiposity, hyperinsulinemia and male-specific hepatic steatosis. To determine the effects of estrogen ablation and subsequent replacement in males on whole body glucose metabolism, three- and six-month-old male ArKO mice were subjected to whole body glucose, insulin and pyruvate tolerance tests and analyzed for ensuing metabolic changes in liver, adipose tissue, and skeletal muscle. Estrogen-deficient male ArKO mice showed increased gonadal adiposity which was significantly reduced upon 17β-estradiol (E2) treatment. Concurrently, elevated ArKO serum leptin levels were significantly reduced upon E2 treatment and lowered serum adiponectin levels were restored to wild type levels. Three-month-old male ArKO mice were hyperglycemic, and both glucose and pyruvate intolerant. These phenotypes continued through to 6 months of age, highlighting a loss of glycemic control. ArKO livers displayed changes in gluconeogenic enzyme expression, and in insulin signaling pathways upon E2 treatment. Liver triglycerides were increased in the ArKO males only after 6 months of age, which could be reversed by E2 treatment. No differences were observed in insulin-stimulated *ex vivo* muscle glucose uptake nor changes in ArKO adipose tissue and muscle insulin signaling pathways. Therefore, we conclude that male ArKO mice develop hepatic glucose intolerance by the age of 3 months which precedes the sex-specific development of hepatic steatosis. This can be reversed upon the administration of exogenous E2.

## Introduction

The vital role of estrogen balance in male metabolic regulation has been highlighted as both men with aromatase deficiency and aromatase knockout (ArKO) mice develop metabolic syndrome (MetS) [1]–[5]. The ArKO mouse, with targeted deletion of exon 9 of the *Cyp19* gene, is an estrogen-deficiency due to its inability to convert androgens into estrogens. The ArKO mouse presents with some of the key factors of MetS - central obesity, hypertriglyceridemia, hyperglycemia, hypo-HDL-cholesterolemia and male-only-hepatic steatosis [5].

When adipose tissue exceeds its storage capacity, as is witnessed in obesity, other peripheral tissues have the potential to begin to increase storage of excessive non-esterified fatty acids (NEFA) [6]. Increased plasma NEFA inhibit glucose utilization, stimulate gluconeogenesis [7], [8] and increase inflammation. Ectopic lipid accumulation in the liver (hepatic steatosis) has been proposed as a link between the development of obesity and insulin resistance [9].

A key element in maintaining glucose homeostasis is the control of liver gluconeogenesis by insulin and glucagon [10]. Gluconeogenesis is reliant on two key enzymes - glucose-6-phosphatase (G6Pase) and phosphoenolpyruvate carboxykinase (PEPCK) [11]. In states of hepatic insulin resistance, these enzymes are not suppressed by insulin and result in elevated endogenous glucose production which contributes to hyperglycemia. Sexually dimorphic differences in the prevalence of hepatic steatosis exist, with significantly higher rates displayed in boys (51.2%) than age matched girls (12.2%) [12] and in white men (42%) versus matched women (24%) [13]. Fat accumulation in the liver in both genders has been associated with insulin resistance and visceral depot size, but only males showed a direct relationship with the steroid hormone profile (free androgen and estrogen index) [12] thereby suggesting that hepatic steatosis may be regulated by sex hormones.

In agreement with human data, we have previously demonstrated that the male ArKO mice suffer from hepatic steatosis which can be rectified upon estrogens treatment [14], [15]. However, the mechanisms behind this have not been studied, emphasizing the need for further investigation into the MetS phenotype of the ArKO mouse.

In this study we provide direct evidence that using the ArKO mouse model, which has no aromatase and hence essentially zero levels of estrogens, allowed us to investigate the effects of exogenous estrogens on the management of glucose metabolism without the interference of endogenous estrogen production and determined that estrogen deficiency leads to hepatic glucose intolerance which precedes the development of male-specific hepatic steatosis.

## Materials and Methods

All efforts were made to minimize animal suffering and procedures were approved by the Monash Medical Centre Animal Ethics Committee (Permit Number: MMCB2008/08)

### Mice

Aromatase Knockout (ArKO) mice (C57Black6 X J129) were generated by disruption of the *Cyp19* gene [16]. Homozygous null or wild-type (*WT*) offspring were bred by crossing heterozygous ArKO and were genotyped by PCR [17]. Mice were housed in groups under pathogenic free conditions, fed a soy-free mouse chow (Glen Forest Stock feeders, Perth, Australia) and water *ad libitum* as previously described [17].

### Treatment and tissue collection

#### Estrogen and placebo treatment

Male mice of both ArKO and WT at 3 and 6 months of age were used in these studies, groups included were: untreated WT, untreated ArKO and ArKO implanted 17β-estradiol pellet (E2). Three month-old ArKO mice were implanted with E2 pellet (E2) (0.05 mg in 21 days i.e. 2.5 µg/day, Innovative Research of America, Toledo, OH, USA) for 3 weeks. Six month-old ArKO mice were implanted with E2 pellet (0.15 mg in 60 days, i.e. 2.5 µg/day; Innovative Research of America, Toledo, OH, USA) 6 weeks. In preliminary studies no difference in body mass or glucose tolerance were detected between untreated and placebo pellet (saline 60 day slow release, Innovative Research of America, Toledo, OH, USA) treated six month-old ArKO male mice (Table S1 and Figure S1). A total number of 5–15 mice were used per group and age, details specified on related graphs. After treatment, mice were killed using a lethal dose of anesthetic (Ketamine (300 mg/kg)/Xylazine (30 mg/kg)). Blood was collected by cardiac puncture and serum was separated, and stored at −20°C. Adipose, liver and muscle tissues were removed, weighed, snap frozen and stored at −80°C.

#### Insulin stimulated tissue collection

After overnight fasting, mice were anaesthetized (6 mg pentobarbital sodium/100 g body weight; Sigma, St Louis USA), and injected with a bolus of insulin (150 U/kg, Actrapid; Novo Nordisk, Bagsvaerd, Denmark) via the descending branch of the inferior vena cava. After 5 min, skeletal muscles (gastrocnemius, extensor digitorum longus, tibialis anterior and soleus), liver and gonadal fat were excised and rapidly frozen in liquid nitrogen for storage at −80°C. Fasting blood samples were collected, serum separated and stored at −20°C.

### Tolerance tests

All mice cohorts were subjected to glucose, insulin and pyruvate tolerance tests, with approximately five days of recovery between each test; glucose tolerance test (GTT – 1 g glucose/kg of body weight, after 8 h of fasting), insulin tolerance test (ITT – 0.5U insulin/kg of body weight, after 8 h of fasting) and pyruvate tolerance test (PTT- 1 g pyruvate/kg of body weight, after ∼16–20 h of fasting). Blood samples were obtained by tail bleeding and analyzed for glucose content (AccuChek Performer, Roche, Mannheim, Germany) immediately before and at 20, 40, 60, 90 and 120 min after an intraperitoneal injection.

### *Ex-vivo* muscle glucose uptake assay

Soleus and extensor digitorum longus (EDL) muscles from WT and ArKO mice were dissected from anesthetized 6 month-old male mice and *ex vivo* glucose uptake was measured with or without insulin (2.8 nM Actrapid; Novo Nordisk, Bagsvaerd, Denmark) as previously described [18]. Muscle tracer uptake was measured by liquid scintillation counting (Tri-Carb 2000, Packard Instrument) of the muscle lysate/supernatant which was prepared by homogenization of the tissue sample in ice-cold protein lysis buffer (50 mM HEPES, pH 7.4, 150 mM NaCl, 10 mM NaF, 1 mM sodium pyrophosphate, 0.5 mM EDTA, 250 mM sucrose, 1 mM DTT, 1 mM Na_3_VO_4_, 1% Triton X-100 and 1 Roche protease inhibitor tablet per 50 ml) followed by centrifugation at 4°C, 13,000 rpm for 30 min and collected.

### Insulin assay

Insulin was measured by ELISA (#EZRMI-13K, Linco Research, St. Charles, MO, USA) following manufacturers' protocol. Briefly, a 96-well microtiter plate pre-coated with monoclonal mouse anti-rat insulin antibody was incubated with 10 µl of control, standards or sample serum plus a biotinylated anti-insulin antibody horseradish peroxidase and a light sensitive 3,3′,5,5′ tetramethylbenzidine substrate allowing enzyme activity to be measured at 450 nm on the Envision Plate reader v 1.09 (Perkin Elmer, Waltham, MA, USA). HOMA-IR was calculated using the following formula: HOMA-IR  =  fasting glucose (mmol/l) × fasting insulin (µU/l)/22.5.

### Protein isolation

Samples were homogenised in ice-cold protein lysis buffer (2.5 mM HEPES, 68.5 mM NaCl, 0.5 mM MgCl2, 0.5 mM CaCl2, 5 mM NaF, 1 mM EDTA, 5 mM Na pyrophosphate, 1 mM NaVO4, 0.5% Nonidet P40 and 5% glycerol) containing protease inhibitors (Complete Mini; Roche, Mannheim, Germany), then incubated on ice for 45 min. Samples were then centrifuged for 15 min at 14,000 *g* before assay of supernatants for protein content by the bicinchoninic acid (BCA) method (Pierce Biotechnology, Rockford, IL, USA)

### Western analysis

Isolated protein (50 µg) together with 1X sample loading buffer (0.125 M Tris-Cl, 4% SDS, 20% v/v Glycerol, 0.2 M DTT, 0.02% Bromophenol Blue, pH 6.8) was boiled for 5 mins at 95°C before SDS-PAGE analysis (10% polyacrylamide resolving gel and 4% stacking gel). Samples were transferred to a nitrocellulose membrane (Amersham™ GE Healthcare, Piscataway USA). Primary antibodies diluted in 1% BSA in Tris Buffered Saline with 1% Tween were incubated overnight at 4°C (mouse anti-Phosphor-Akt (Ser473), rabbit anti-Akt, rabbit anti-phosphor-AMPK (Thr172), rabbit anti-AMP alpha (Cell Signaling Technologies, Beverly MA, USA). Appropriate Alexa Fluor secondary antibodies were incubated at room temperature for 1 h. Protein band intensities were quantified using the Odyssey infrared imaging system (Licor Biosciences, Lincoln, NE, USA). All protein quantifications were normalized to housekeeping protein β tubulin (mouse- anti- β tubulin; Millipore, California USA).

### RNA isolation and real-time PCR

Total RNA was extracted from WT, ArKO and ArKO + E2 mouse frozen liver tissue using Ultraspec RNA isolation system (Fisher Biotec, WA, Australia) following manufacturer's protocol. All samples were DNase I treated (DNAfree™, Ambion, Foster City, CA, USA) and concentrations were quantified using the NanoDrop®1000 (Thermo, Willmington, USA). Total RNA (1 µg) was reverse transcribed (Roche, Mannheim, Germany) and amplified by real-time PCR using a SYBR ‘Master Mix’ (DMSO (Sigma-Aldrich, USA) 10X Gold PCR Buffer, AmpliTaqGold (Applied Biosystems, California, USA), 1 M Mg(oAC)2 (Sigma-Aldrich, USA), 100 mM dACT, dCCT, dGCT and dTCT (Bioline, Australia) 1 µg/µl 6-ROX and 10,000X con SYBR (Invitrogen, USA)) in the ABI7900H (Applied Biosystems, California, USA) with specific oligonucleotide pairs (see Table 1). Real-time PCR data were calculated using ΔΔCT method of analysis. All samples were normalized to housekeeping gene cyclophilin transcripts levels.

**Table 1:** Real-time PCR primers.

Transcript	Oligonucleotide pairs (5′-3′)	Amplicon Size (bp)
Glucose-6-phosphatase (G6Pase)	For: TCTTGTGGTTGGGATACTGG	110
	Rev: AGCAATGCCTGACAAGACTC	
Phosphophenolpyruvate carboxykinase (PEPCK)	For: GTGTCCCCCTTGTCTATGAA	163
	Rev: GGTATTTGCCGAAGTTGTAG	
Insulin Receptor Substrate 1 (IRS1)	For;CAGCGATGGCGAAGGCACCA	272
	Rev;GCGAACCGGACACGGAAGCA	
Insulin Receptor Substrate 2 (IRS2)	For: GCGGCCTCATCTTCTTCACT	129
	Rev: AACTGAAGTCCAGGTTCATATA	
Glycogen Substrate Kinase 3 beta (GSK3β)	For: ACCAATATTTCCTGGGGACA	130
	Rev: GTACCTTGATTTGAGGGAAT	
Fatty Acid Synthase (Fasn)	For: CACAGATGATGACAGGAGATGGA	205
	Rev: TCGGAGTGAGGCTGGGTTGATA	
Stearoyl CoA desaturase 1 (Scd-1)	For: CATCATTCTCATGGTCCTGCT	236
	Rev: CCCAGTCGTACACGTCATT	
Acetyl-CoA Carboxylas (ACCα)	For: TGTTGGGGTTATTTCAGTGTTGC	236
	Rev: TGTCCAGCCAGCCAG GTCG	
Cyclophilin	For: CTTGGGCCGCGTCTCCTTC	180
	Rev: TGCCGCCAGTGCCATTAT	

List of primer sequences (and expected amplicon sizes) used for Real-Time PCR.

### Liver triglyceride (TG) content

Saponified extracts were prepared from frozen liver samples, and the triglyceride content was quantified by comparing to a glycerol standard curve, as previously described [19], [20]. Briefly, hepatic triglycerides (TG) were extracted from 100–300 mg liver tissues overnight at 55°C in ethanolic potassium hydroxide, and neutralized with water:ethanol (1∶1 vol/vol). Saponification proceeded with 1 M MgCl_2_ by vortexing and incubating at 4°C. Saponified liver extracts (the upper phase) were separated by centrifugation, and the glycerol content was quantified with Free Glycerol Reagent (Sigma-Aldrich, Missouri, USA), which generate a dye with an absorbance at 540 nm (measured using the Wallac 1420 Victor Plate Reader, LabX, Midland ON Canada). All blanks, glycerol standards and samples were quantified in duplicates.

### Adipokine panels

Adipokine levels in mouse serum were analyzed using the adipokine LINCOplex Kit 96 well assay according to manufacturer's protocol (Mouse Serum single-plex adiponectin panel #MADPK-71-ADPN; Mouse Serum Adipokine kit #MADPK-71K – Leptin; Millipore, California USA). Briefly, standards, quality controls and serum samples were pipetted in duplicate in a 96-well microtiter filter assay plate and incubated overnight at 4°C with antibody immobilized beads. Mouse adipokine detection antibodies were added and incubated. Unhybridized antibodies were removed and Streptavidin-Phycoerythrine incubated. Luminex sheath fluid was added; plates were scanned and analyzed on the Luminex® 100 instrument and software (Luminex Corporation, Texas, USA).

### Statistical analysis

All statistical analyses were performed using GraphPad Prism® version 5 for windows (GraphPad Software Inc). WT untreated, ArKO untreated and ArKO + E2 treatment groups were analyzed using a two-tailed Mann-Whitney test. Data are expressed as mean ± standard deviation (SD).

## Results

### Effects of estrogens on body and gonadal adipose tissue weights

There were no differences between male WT and ArKO mice in body weight at both ages (3 and 6 month-old; Table 2). Placebo treatment in ArKO mice did not induce any changes in body weight or gonadal adipose tissue weight (Table S1). However upon 17β-estradiol (E2) treatment, body weights of ArKO mice at 3 and 6 months were reduced (p<0.05 and p<0.0001 vs untreated KO respectively; Table 2), and beyond that of the WT at 6 months (p<0.01 vs WT). In a consistent pattern, gonadal adipose tissue weights were increased in the 3 and 6 month-old untreated ArKO compared to that of WT (p<0.01 and p<0.05 respectively), and again reduced upon E2 treatment (p<0.001 and p<0.0001 vs untreated KO respectively).

**Table 2:** Body and adipose weights, adipokines levels.

	WT	KO	KOE
**Body weight (g)**			
3mth	25.50±2.71	30.03±6.66	25.21±2.73^#^
6mth	36.71±6.14	37.12±3.99	29.45±3.72**^###^
**Gonadal adipose (g)**			
3 mth	0.37±0.11	0.91±0.47**	0.31±0.10^###^
6 mth	1.05±0.52	1.69±0.33*	0.69±0.32^###^
**Leptin (ng/ml)**			
6 mth	5.93±0.78	11.79±7.9*	3.85±1.4*^##^
**Adiponectin (µg/ml)**			
6 mth	9.87±1.04	7.38±1.53*	9.61±3.55

Body and gonadal adipose tissue weights in grams (g) and serum leptin in micrograms per milliliter (µg/ml) and adiponectin levels in nanograms per milliliter (ng/ml) of 3 and 6 month-old male wild type (WT), aromatase knockout (KO) and 2.5 µg/day 17β-estradiol-treated KO (KOE). Data are expressed as mean ± SD; n = 7–8 per group,*p<0.05 and **p<0.01 versus WT mice. ^#^ *p<0.05*, ^##^ *p<0.01* ^###^ *p<0.001* versus KO.

### Serum adiponectin and leptin levels

Six month-old ArKO mice had reduced levels of serum adiponectin (p<0.05; Table 2), which returned to WT levels upon E2 treatment. Conversely, serum leptin levels in the ArKO mice were higher than WT counterparts (p<0.03). E2 administration dramatically reduced serum leptin levels in ArKO mice to below untreated WT controls (p<0.01, Table 2).

### The effects of estrogens on serum insulin and glucose levels

**Figure 1:**
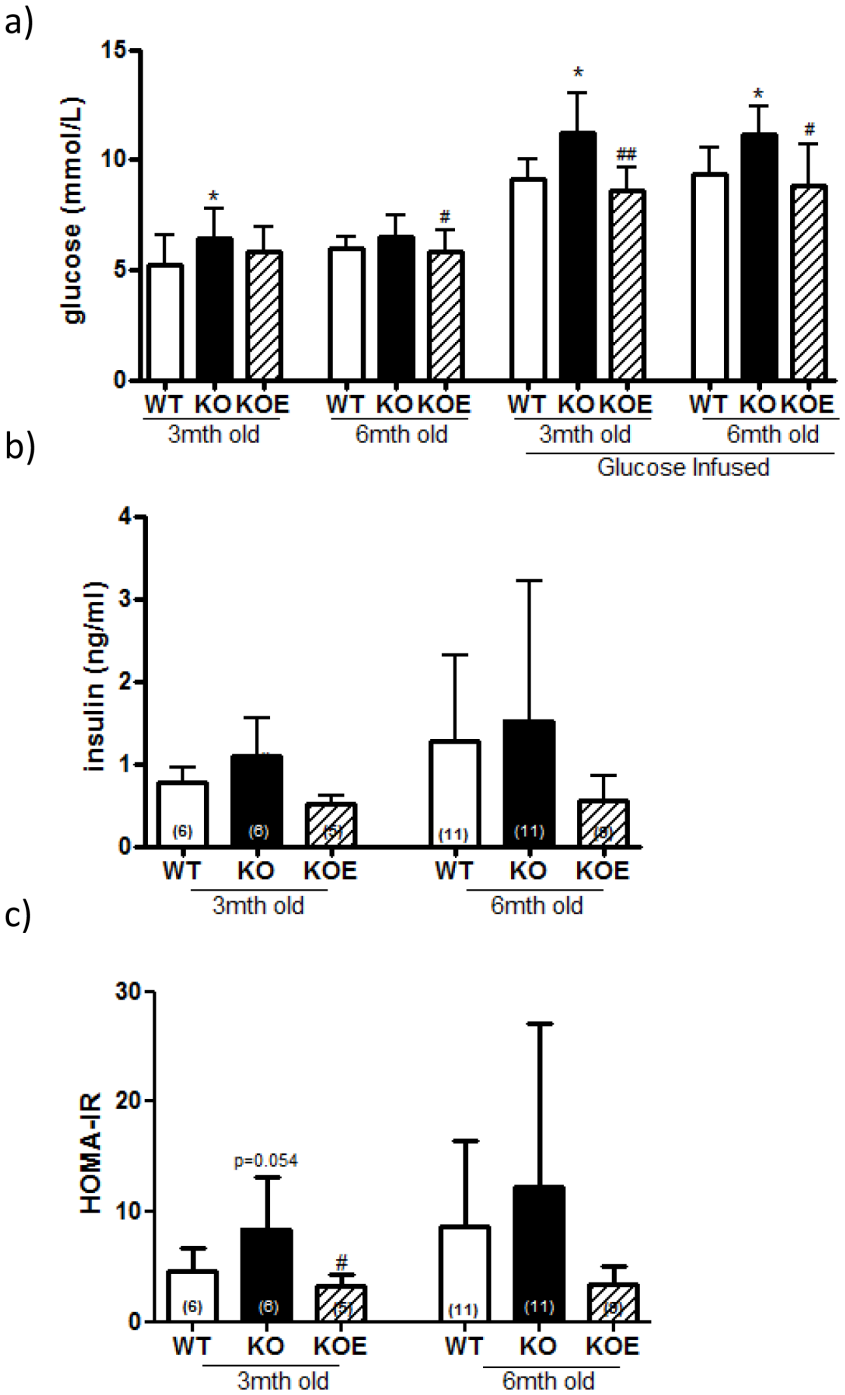
Serum glucose, insulin and HOMA-IR levels. (**a**) Fasted and 20 min post glucose infused serum glucose (**b**) Fasted serum insulin and (**c**) HOMA-IR (fasted serum glucose (mmol/l) × fasted serum insulin (µU/l)/22.5) from 3 and 6 month-old fasted male wildtype (WT) and aromatase knockout (KO) and KO treated with 2.5 µg/day estrogen (KOE). Expression data from samples (n =  shown on corresponding bar) per genotype are shown, and are presented from replicate analysis as the mean ± SD. *p<0.05 versus WT mice. ^#^ *p<0.05* versus KO.

Fasted basal serum insulin and glucose levels of the 3 month-old male ArKO mice were elevated, (n/s and p<0.05 respectively) compared to WT counterparts, a difference which was improved by E2 (2.5 µg/day) treatment (p<0.05 and n/s, Figure 1a, b). HOMA-IR showed a trend for increase between WT and untreated ArKO (p = 0.051) but was significantly reduced in ArKO with E2 treatment (p<0.05). In 6 month-old animals, there were no differences between WT and ArKO untreated fasted basal serum insulin, glucose or HOMA-IR, additionally no changes were seen upon E2 treatment (2.5 µg/day) in the ArKO's (Figure 1a, b, c)

### Whole body glucose, pyruvate and insulin tolerance in the ArKO mice

Whole body tolerance tests were performed on WT, ArKO and E2 administrated mice at 3 and 6 months of age. No differences were seen in glucose tolerance between untreated and six-week placebo treated ArKO mice (Figure S1).

#### Three month-old animals

**Figure 2:**
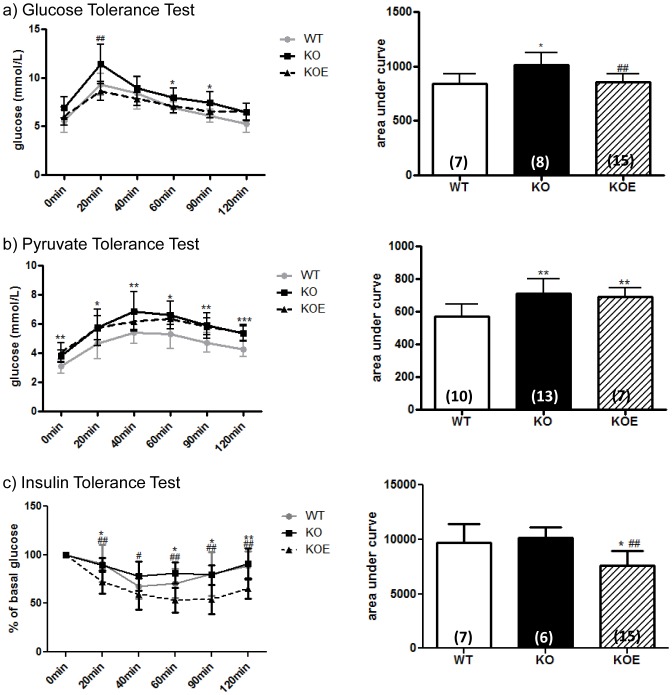
Three month-old male whole body glucose, insulin and pyruvate tolerance. Whole body tolerance tests were completed on fasted three month-old male wildtype (WT) and aromatase knockout (KO) and 2.5 µg/day 17β-estradiol-treated KO (KOE) (**a**) glucose tolerance test and corresponding area under curve; (**b**) insulin tolerance test and corresponding area under curve; (**c**) pyruvate tolerance test and corresponding area under curve Data are presented from replicate analysis (n =  shown on corresponding bar) as the mean ± SD. *p<0.05, **p<0.01, ***p<0.001, versus expression in age-matched WT samples and *^#^p<0.05 and ^##^p<0.01* versus KO samples.

Three month-old ArKO mice exhibited lower glucose and pyruvate tolerance compared to WT littermates (p<0.05 and p<0.01 respectively, Area Under Curve (AUC), Figure 2a, b), highlighting a loss of glycemic control. After 3 weeks of E2 treatment (2.5 µg/day), glucose tolerance and insulin sensitivity were improved (p<0.01 and p<0.01 respectively, AUC) as compared to the untreated ArKO. Further, insulin sensitivity with E2 treatment in the ArKO lowered below that of the WT littermates (p<0.05; Figure 2a, c).

#### Six month-old animals

**Figure 3:**
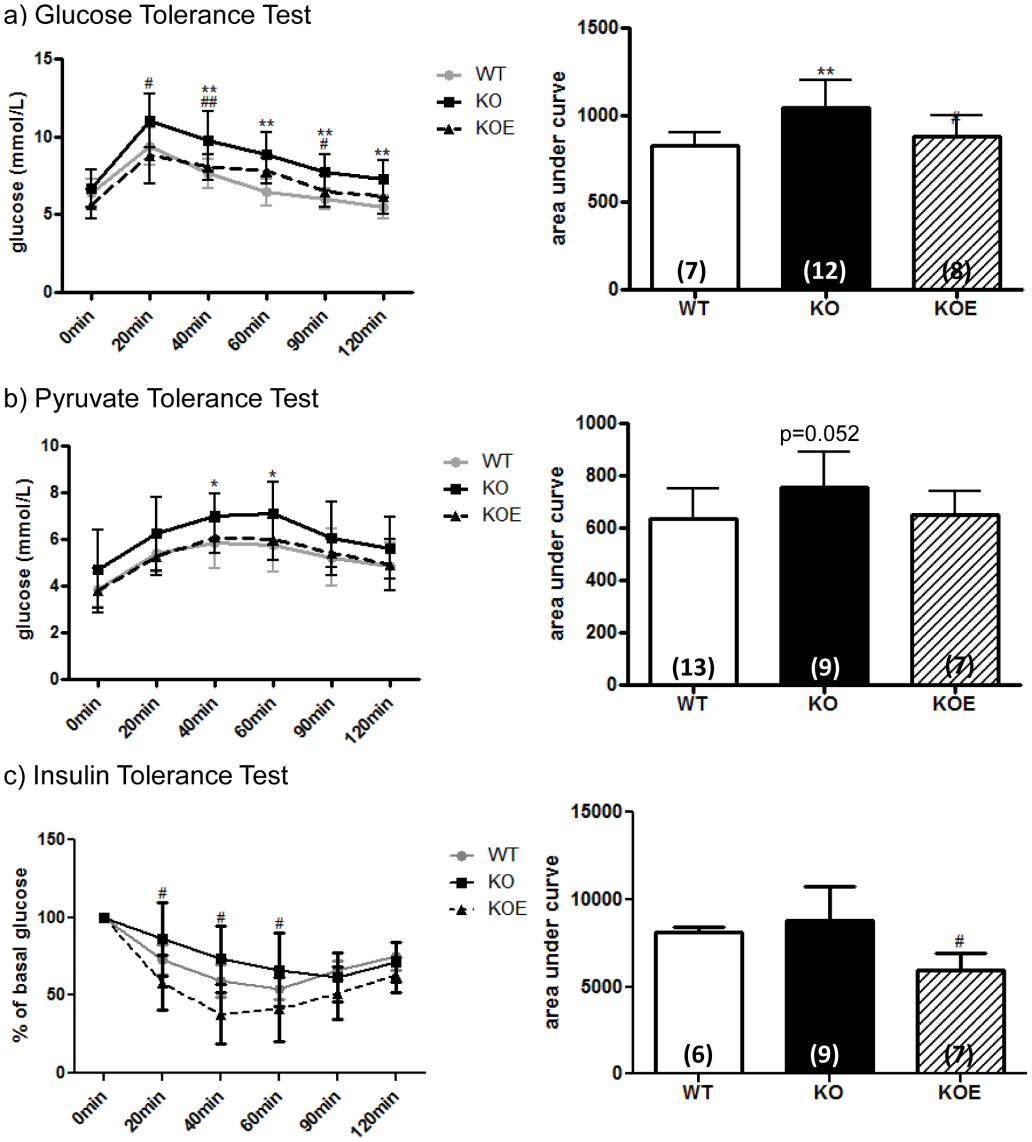
Six month-old male whole body glucose, insulin and pyruvate tolerance. Whole body tolerance tests were completed on fasted 6 month-old male wildtype (WT), aromatase knockout (KO) and 2.5 µg/kg 17β-estradiol-treated KO (KOE). (**a**) Glucose tolerance test and corresponding area under curve; (**b**) insulin tolerance test and corresponding area under curve; (**c**) pyruvate tolerance test and corresponding area under curve. Data are presented from replicate analysis (n =  shown on corresponding bar) as the mean ± SD. *p<0.05, **p<0.01, versus expression in age-matched WT samples and *^#^p<0.05 and ^##^p<0.01* versus KO samples

Six month-old ArKO mice displayed impaired glucose tolerance (p<0.01, AUC) and signs of pyruvate intolerance (p = 0.052, AUC) as compared to WT littermates (Figure 3a, b). The administration of E2 (2.5 µg/day) for 6 weeks to ArKO mice markedly improved glucose tolerance (p<0.05, AUC) and insulin sensitivity (p<0.05, AUC) As seen in the 3-month old mice, E2 treatment in the ArKO mouse was able to reduce insulin sensitivity below levels observed in WT littermates (Figure 3a, c).

### Liver triglycerides in the male *ArKO*

**Figure 4:**
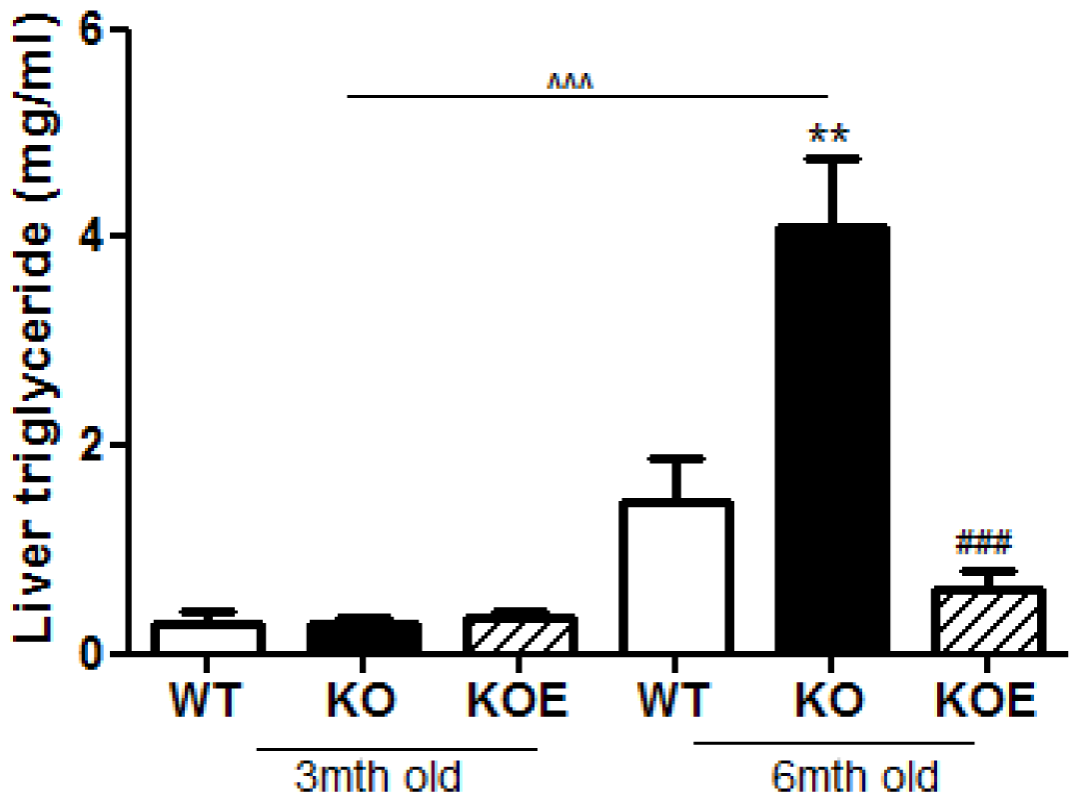
Liver Triglyceride levels. Liver triglyceride assay were performed on 3 and 6 month-old fasted male wildtype (WT), aromatase knockout (KO) and 17β-estradiol-treated KO (KOE) mice. Expression data from 7–8 samples per genotype are shown following and presented from replicate analysis as the mean ± SD. **p<0.01, versus expression in age-matched WT samples and *^###^p<0.001* versus age-matched KO samples and ∧∧p<0.01, ∧∧∧p<0.001, versus 3mth old KO samples.

In 3 month old animals, there were no differences in liver triglycerides levels between WT, ArKO and ArKO+E2 mice. In 6 month old animals, liver triglycerides were substantially increased in ArKO compared with WT animals (p<0.01) and E2 treatment reduced triglycerides to below WT levels (p<0.001; Figure 4). These 6 month-old ArKO mice also had significantly (p<0.001) higher triglyceride levels compare to the 3 month-old ArKO male mice (Figure 4).

### *Ex-vivo* muscle glucose uptake

After observing signs of glucose dysregulation in the untreated ArKO male mice, we investigated *ex vivo* insulin stimulated muscle glucose uptake in 6 month-old mice. Surprisingly, there were no differences in insulin-stimulated glucose uptake in EDL or soleus muscle between the untreated ArKO mice and their WT littermates (Figure S2a, b). These data suggest that whole-body glucose intolerance in ArKO mice is not due to defects in skeletal muscle insulin-sensitivity.

### Gonadal adipose tissue, liver and muscle protein phosphorylation

**Figure 5:**
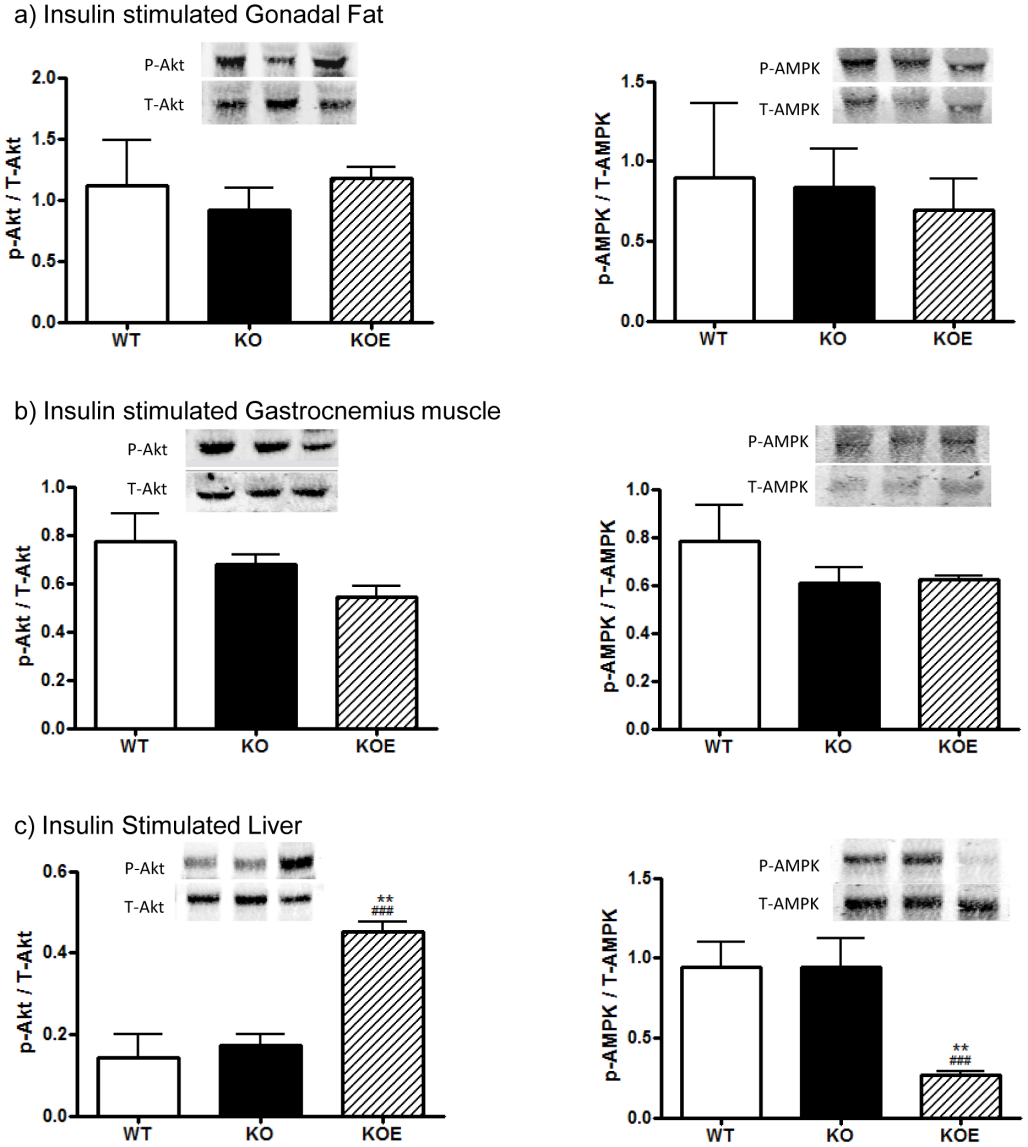
Western blot analyses of insulin stimulated and basal tissue. Protein phosphorylation analyses of Akt and AMPK levels were performed on protein extracted from insulin stimulated (**a**) gonadal adipose tissue, (**b**) gastrocnemius muscle, (**c**) liver in insulin stimulated liver of 6 month-old male wildtype (WT) and aromatase knockout (KO) and 2.5 µg/day estrogen-replaced KO (KOE) mice. Expression data from 6–8 samples per genotype are shown, and presented from replicate analysis as the mean ± SD. *p<0.05, **p<0.01, ***p<0.001 versus expression in age-matched WT samples and *^#^p<0.05, ^###^p<0.001* versus KO samples.

To determine the potential mechanism responsible for differences in glucose intolerance between WT and ArKO mice, the expression levels and phosphorylation of Akt and AMPK, which plays a role in insulin mediated glucose uptake, in gonadal adipose tissue, skeletal muscle and liver were analyzed. In 6 month-old untreated ArKO mice, there were no changes in the phosphorylation of Akt or AMPK, normalized to total protein, in investigated adipose, muscle and liver tissues compared to WT mice (Figure 5a, b, c). However, with 6 weeks of E2 treatment (2.5 µg/day), there was a clear increase in liver Akt phosphorylation and decrease in AMPK phosphorylation compared to both the untreated ArKO (p<0.001 and p<0.001 respectively) and WT counterparts (p<0.01 and p<0.01 respectively; Figure 5c).

### Insulin signaling pathway mRNA expression levels in the liver

After witnessing significant changes in the liver phenotype but not gonadal adipose tissue or skeletal muscle, the regulation of hepatic glucose homeostasis was investigated.

**Figure 6:**
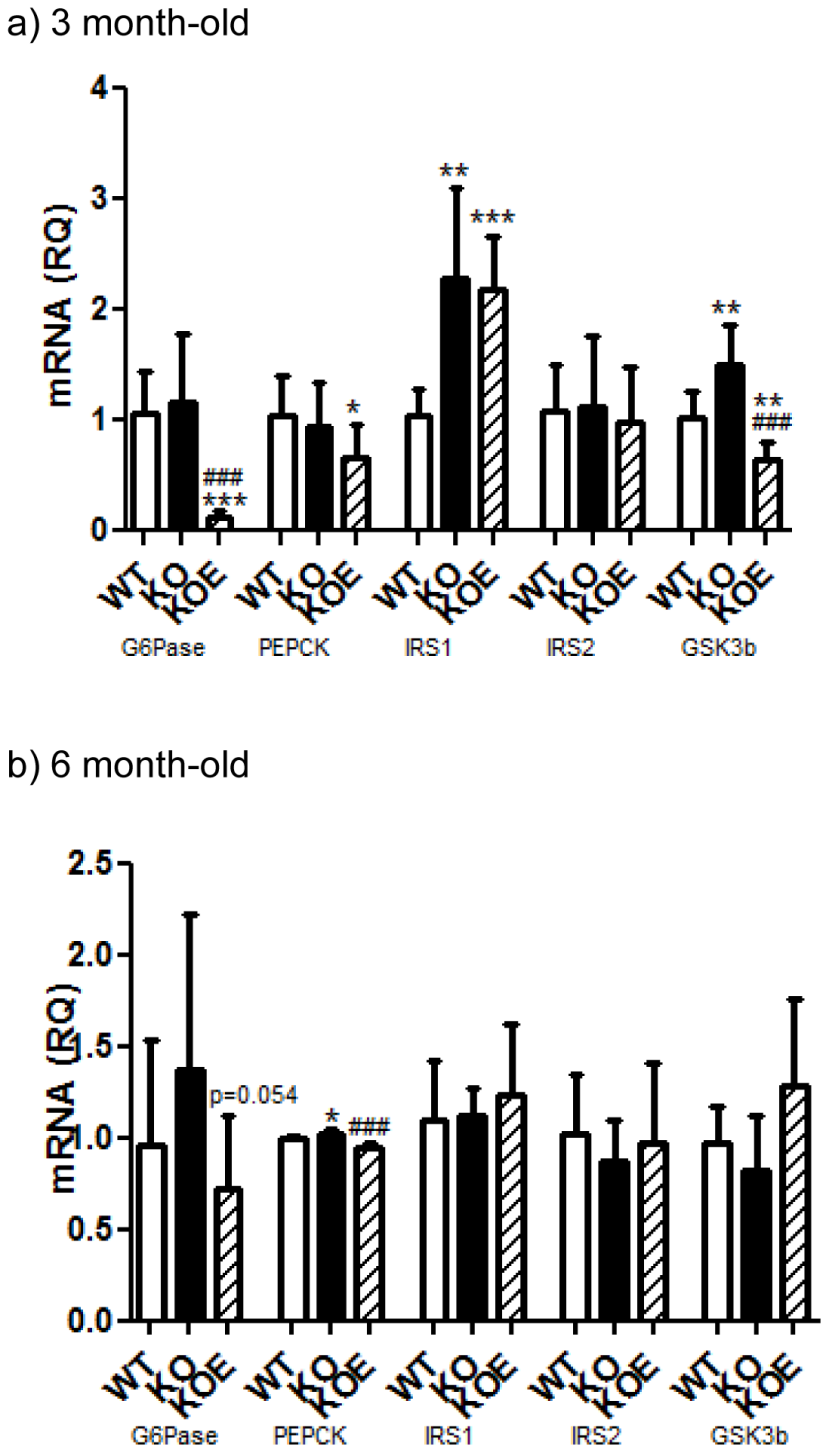
Real-time PCR analysis of gluconeogenesis mRNA expression in the liver. Real-time-PCR analyses of G6Pase, PEPCK, IRS1, IRS2 and GSK3β mRNA expression were performed on cDNA derived from total RNA prepared from fasted liver tissue of (a) 3 and (b) 6 month-old male wildtype (WT, n = 8), aromatase knockout (KO, n = 8) and 2.5 µg/day 17β-estradiol-treated KO (KOE, n = 7) mice. Expression data from 7–8 samples per genotype are shown following normalization for cyclophilin mRNA expression, and presented from replicate analysis as the mean ± SD. *p<0.05,**p<0.01 and ***p<0.001 versus expression in age-matched WT samples and *^##^p<0.01, ^###^p<0.05* versus KO samples.

In the 3 month-old mice both GSK3 β and IRS1 mRNA levels were significantly increased (p<0.01 and p<0.01, respectively) in the ArKO compared to WT counterparts. Upon E2 treatment GSK3β mRNA expression was subsequently decreased (p<0.001 vs KO and p<0.01 vs WT), whereas IRS1 mRNA levels remained elevated with no improvement compared to untreated ArKO. Estrogen treatment was also able to significantly reduce G6Pase and PEPCK mRNA levels (p<0.001 and p<0.05 respectively) in the ArKO mouse. There was no difference in the expression levels of IRS2 (Figure 6a).

Consistent with differences in pyruvate tolerance, fasted 6 month-old ArKO male mice liver transcript levels of PEPCK (Figure 6b) were significantly increased (p<0.05) compared to WT. Upon E2 treatment, G6Pase and PEPCK gene expressions are both decreased (p = 0.054 and p<0.001 respectively) compared to untreated ArKO. However, neither ArKO untreated nor E2 treatment had any effects on GSK3β, IRS1 and IRS2 expression levels (Figure 6b).

### Lipid profile mRNA expression levels in the liver

**Figure 7:**
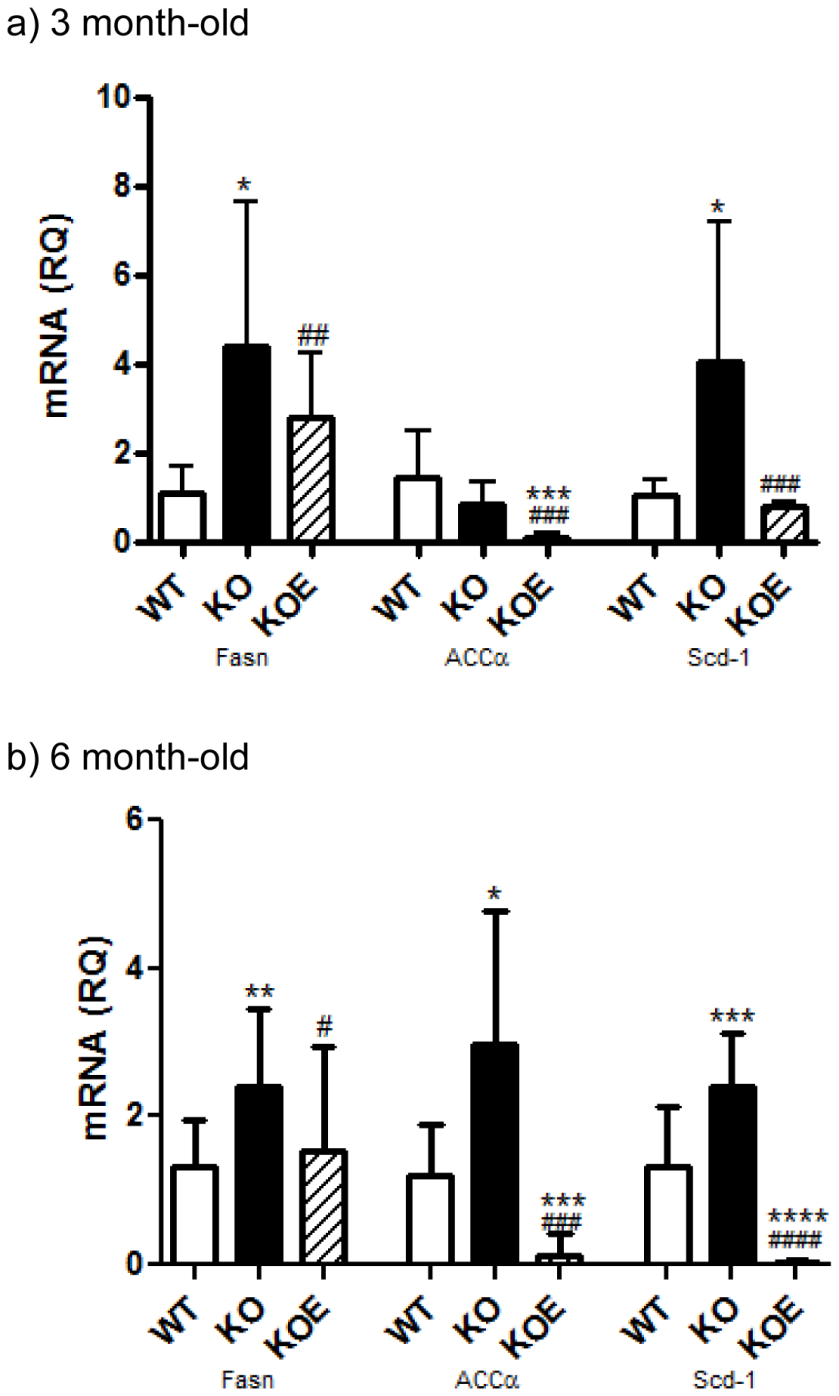
Real-time PCR analysis of mRNA lipid profile in the liver. Real-time-PCR analyses of Fasn, ACCα and Scd-1 mRNA expression were performed on cDNA derived from total RNA prepared from fasted liver tissue of (a) 3 and (b) 6 month-old male wildtype (WT, n = 8), aromatase knockout (KO, n = 8) and 2.5 µg/day 17β-estradiol-treated KO (KOE, n = 7) mice. Expression data from 7–8 samples per genotype are shown following normalization for cyclophilin mRNA expression, and presented from replicate analysis as the mean ± SD. *p<0.05, **p<0.01 and **p<0.001, versus expression in age-matched WT samples and *^##^p<0.01, ^###^p<0.001* versus KO samples.

Fasn, and Scd-1 mRNA levels were increased in the ArKO liver compare to the WT counterparts in the 3month-old (p<0.05 and p<0.01 respectively) and 6month-old (n/s and p<0.05 respectively) mice. Estrogen treatment was able to reverse these increases in all cases. ACCα transcript levels were significantly increased (p<0.05) in the 6 month-old ArKO only and not 3 month-old ArKO. However, both 6 and 3 month-old mice had reduced ACCα mRNA when administered E2 (p<0.001vs both WT and KO respectively) (Figure 7a, b).

## Discussion

This study presents data on whole body glucose tolerance in the estrogen deficient male ArKO which is consistent with other studies [21], and provides additional and new insight into the specific actions of key peripheral tissue (liver, adipose and muscle) involved in insulin signalling. Male ArKO mice displayed glucose intolerance at 3-and 6-months of age which could be primarily attributed to elevated hepatic gluconeogenesis as indicated by the results of pyruvate tolerance tests. In agreement with this conclusion, livers from ArKO mice had impaired insulin-stimulated Akt phosphorylation and increased expression of gluconeogenic and lipid biosynthesis enzymes. In contrast, in the absence of estrogens, adipose tissue and muscle insulin sensitivity were unchanged in terms of insulin stimulated Akt and AMPK phosphorylation and *ex-vivo* glucose uptake, despite the expected changes in adipokine levels (lowered adiponectin and elevated leptin levels) with increased body weight and fat deposits in ArKO mice. Interestingly, male ArKO mice did not display a significant increase in liver triglycerides until 6 months of age, thus demonstrating that hepatic glucose intolerance precedes hepatic steatosis in these mice. Importantly, we demonstrated that E2 replacement can improve most aspects of this phenotype.

The intricate ‘cause and effect’ relationship between hepatic steatosis and insulin resistance remains a subject of controversy and research. It has been reported that men have higher rates of insulin resistance which may be related to greater visceral and hepatic adiposity in conjunction with the lower levels of estrogens [22]. The hepatic steatosis phenotype in ArKO mice has also been partially attributed to increases in *de novo* lipogenesis [14] and impaired β-oxidation [23], which can be ameliorated upon estrogen treatment [14]. Accumulation of lipids within the liver may lead to reduced insulin signalling, impaired GLUT translocation and inflammation and is independently linked to type II diabetes [24]–[28]. However, it is also suggested that hepatic steatosis is secondary to an insulin resistant state [9] due to a lack of insulin dependent suppression of lipid synthesis. Our current detection of glucose intolerance without concomitant signs of hepatic steatosis in 3 month-old ArKO mice, followed by hepatic steatosis presentation at 6 months of age, supports this hypothesis.

Our investigation of the lipid profile in the male ArKO mouse liver revealed that mRNA expression of genes involved in fatty acid (FA) synthesis; Fasn (an enzyme involved in the synthesis of palmitate) and Scd-1 (a catalyst of palmitate to unsaturated FA) were already increased by 3 months of age continuing into 6 months. However, ACCα (which provides the malonyl-CoA substrate for FA synthesis) mRNA levels were significantly increased in the 6 month old animals but not in the 3 month old animals. Thus, the resultant translational and physiological effects of increased liver triglycerides and hepatic steatosis do not occur at 3 months of age but at a later age.

Other models of reduced estrogen activity such as the estrogen receptor α knockout (ERαKO) mouse have also confirmed the role of estrogens in disrupted hepatic glucose homeostasis [29], [30], although these experiments have only been published in female mice to date [31], [32]. Furthermore, estrogen administration to *ob/ob* mice reduced hepatic insulin resistance and decreased expression of genes involved in hepatic lipid biosynthesis [33] and improved liver dysfunction parameters [34].

In ArKO mice, estrogens' role in hepatic glucose intolerance may be explained by its ability to significantly reduce key hepatic insulin signaling genes G6Pase and PEPCK upon E2 administration, which we observed by 3 months of age and continued through to 6 months of age. These findings support previous studies which demonstrate direct inhibitory effects of E2 on PEPCK expression via PGC1α (peroxisome proliferator activated receptor gamma co-activator 1 alpha) [35], [36] and studies showing estrogens administration to *ob/ob* mice reduces G6Pase expression [37]. GSK3β was elevated in the ArKO compared to WT and reduced upon E2 treatment at 3months, but not evident at 6 months of age. GSK-3β impairs the ability of insulin to activate glucose disposal and over-expression is associated with insulin resistance. Additionally, the increases seen in IRS1 at 3 months of age (not seen at 6 months) and the lack of recovery upon estrogen treatment at this age also correlated with the lack of improvement in pyruvate tolerance. Furthermore, Kim et.al recently defined the timed dosing effects of estrogen and concluded that short term E2 treatment improved glucose homeostasis via hepatic gluconeogenesis rather than insulin sensitivity in the muscles [38].

Glucose uptake and production is regulated by the insulin signaling pathway of which Akt plays a fundamental role. Akt is known to be expressed in insulin sensitive tissue and *Akt2* knockout mice display severe impairment of insulin stimulated whole body glucose disposal [39]. Increased Akt phosphorylation with improved glucose tolerance was observed in the liver of the E2 treated male ArKO mice. Recent studies highlighting specific E2 effects on Akt demonstrated that in ochidectomized male mice with resultant insulin resistance phenotype, showed E2 alone could increase Akt phosphorylation without effecting other parameters whereas combined T+E2 replacement returned whole body insulin sensitivity to normal levels [40]. Akt is also involved in glycogen synthesis, an insulin stimulated system of glucose storage through the phosphorylation and inactivation of GSK-3β. A vital constituent in both cellular and whole-body energy balance preservation is the adenosine monophosphate-activated protein kinase (AMPK). AMPK also plays a role in increasing glucose uptake and β-oxidation in peripheral tissues, as well as decreasing liver glucose production [41]. Unexpectedly, a steep decrease in levels of phosphorylated AMPK was also present in our estrogen-treated animals. Previous studies have identified that insulin stimulated Akt in the heart can induce Ser485/491 phosphorylation of AMPK which inhibits AMPK Thr172 phosphorylation [42], [43]. This may explain the decrease in AMPK Thr172 phosphorylation seen in the insulin stimulated male ArKO mice treated with E2. In summary, our data indicate that the hepatic steatosis in the 6 month old mice is not a result of decreased phosphor-AMPK and the E2 recovery of hepatic steatosis is not mediated through activation of AMPK.

The ArKO mouse model allowed us to observe the effects of estrogen absence on the relationship between the progression and development of glucose intolerance and hepatic steatosis. Additionally, as the ArKO mouse remains estrogen responsive this allows the effects of administered exogenous E2 to be investigated. The absence of estrogens in the male ArKO mice leads to hepatic glucose intolerance from 3 to 6 months of age with consistent improvement upon E2 treatment. This highlights the potential of combined roles of both estrogens and glucose homeostasis in the development of hepatic steatosis in the male ArKO mice.

## Conclusion

This study shows that the absence of aromatase and hence ablation of estrogens causes defects in hepatic glucose homeostasis at a young age preceding the development of hepatic steatosis which is only witnessed in aged male ArKO mice. Exogenous E2 treatment is able to improve both glucose homeostasis and the pursuant hepatic steatosis phenotype.

## Supporting Information

Figure S1 **Untreated vs. placebo treated ArKO mice glucose tolerance test.** Whole body glucose tolerance tests were completed on fasted six month-old male aromatase knockout (KO) and 2.5 µg/day 17β-estradiol-treated KO (KOE) (**a**) glucose tolerance test and (**b**) corresponding area under curve; Data are presented from replicate analysis (n =  shown on corresponding bar) as the mean ± SD.
(PPT)

Figure S2 **Ex-vivo muscle glucose uptake.** *Ex-vivo* muscle glucose uptake assays were performed on 6 month-old fasted male wildtype (WT), or aromatase knockout (KO) mice, treated with insulin or without (basal). Tracer uptake was measured in dissected (**a**) soleus and (**b**) extensor digitorum longus (EDL) muscles. Expression data from n = 8 samples per genotype as the mean ± SD. *p<0.05, **p<0.01 and ***p<0.01.
(PPT)

Table S1 **Body and gonadal tissue weight of untreated versus placebo treated KO mice.** Body and gonadal adipose tissue weights in grams (g) of 6 month-old male aromatase knockout untreated (KO) and placebo-treated KO (KOP). Data are expressed as mean ± SD; n = 3–8 per group.
(PPT)
