# Supplementary figures and images for: Hepatic Glucose Intolerance Precedes Hepatic Steatosis in the Male Aromatase Knockout (ArKO) Mouse
Source: PLoS One. 2014 Feb 10;9(2):e87230. doi: 10.1371/journal.pone.0087230 (PMC3919708; doi:10.1371/journal.pone.0087230)

## Slide 1
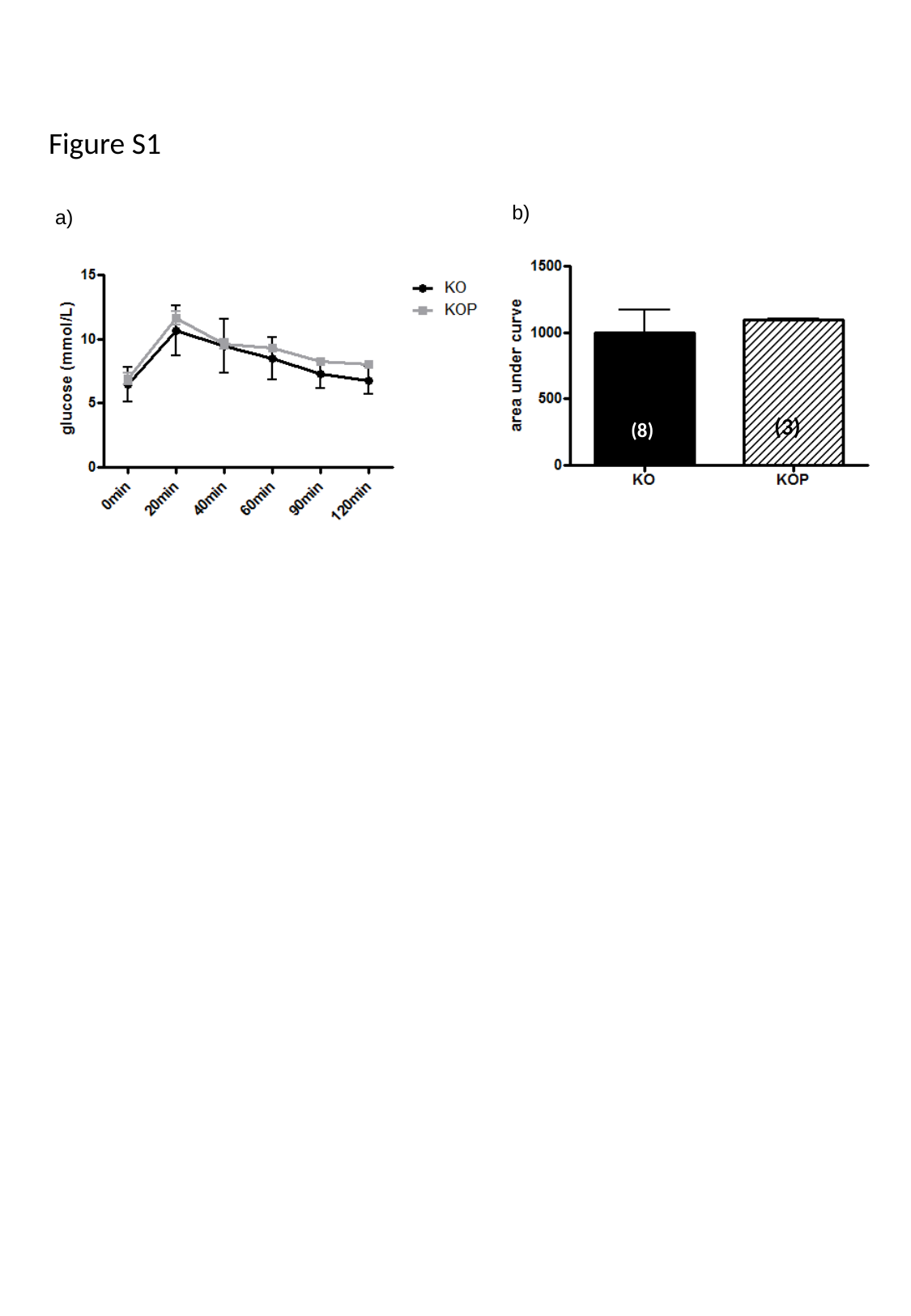

Figure S1
b)
a)
(3)
(8)

Supplement: Figure S1 — Untreated vs. placebo treated ArKO mice glucose tolerance test. Whole body glucose tolerance tests were completed on fasted six month-old male aromatase knockout (KO) and 2.5 µg/day 17β-estradiol-treated KO (KOE) (a) glucose tolerance test and (b) corresponding area under curve; Data are presented from replicate analysis (n = shown on corresponding bar) as the mean ± SD. (PPT) [file pone.0087230.s001.ppt]

## Slide 1
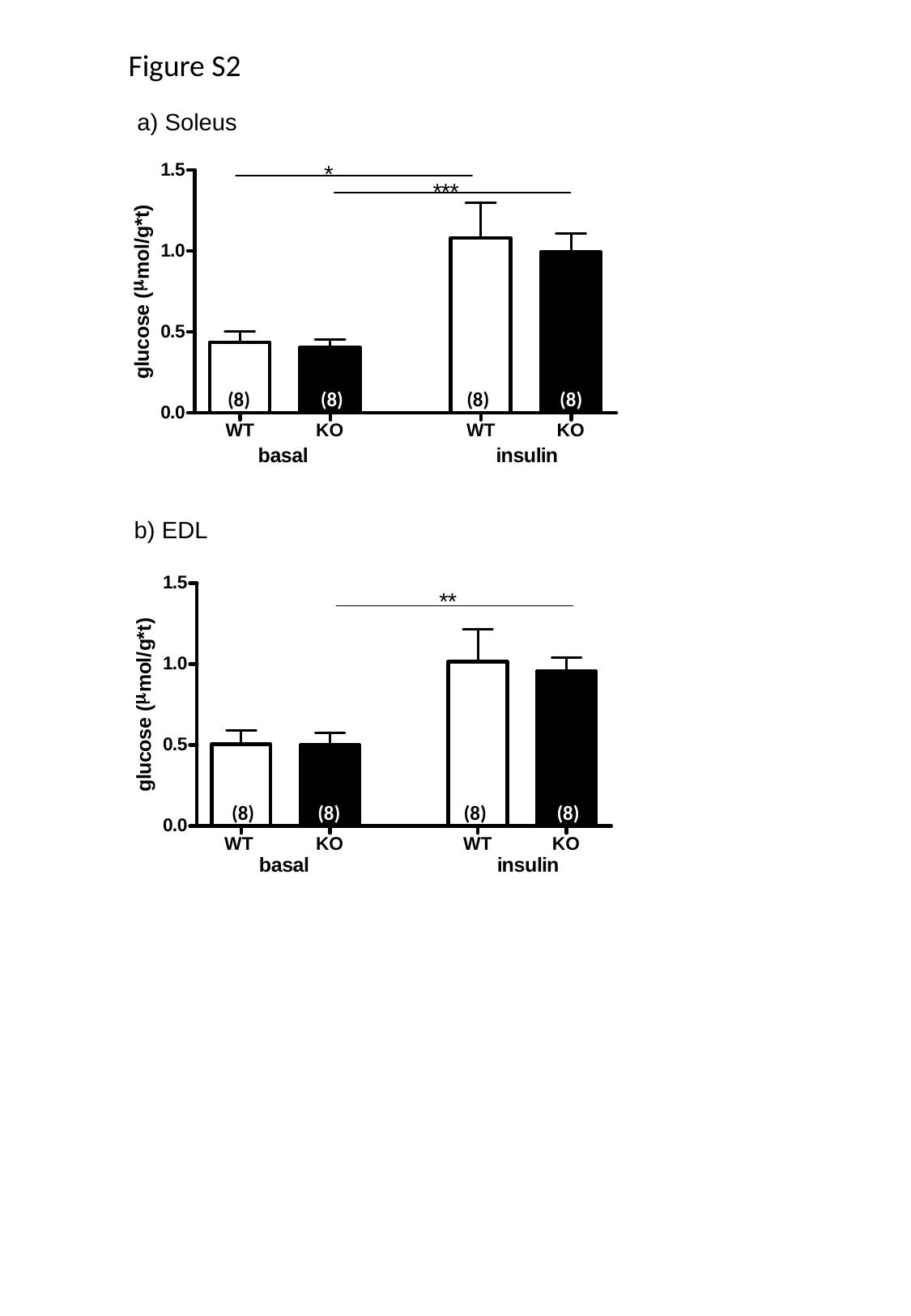

Figure S2
a) Soleus
(8)
(8)
(8)
(8)
b) EDL
(8)
(8)
(8)
(8)
(8)

Supplement: Figure S2 — Ex-vivo muscle glucose uptake. Ex-vivo muscle glucose uptake assays were performed on 6 month-old fasted male wildtype (WT), or aromatase knockout (KO) mice, treated with insulin or without (basal). Tracer uptake was measured in dissected (a) soleus and (b) extensor digitorum longus (EDL) muscles. Expression data from n = 8 samples per genotype as the mean ± SD. *p<0.05, **p<0.01 and ***p<0.01. (PPT) [file pone.0087230.s002.ppt]

## Slide 1
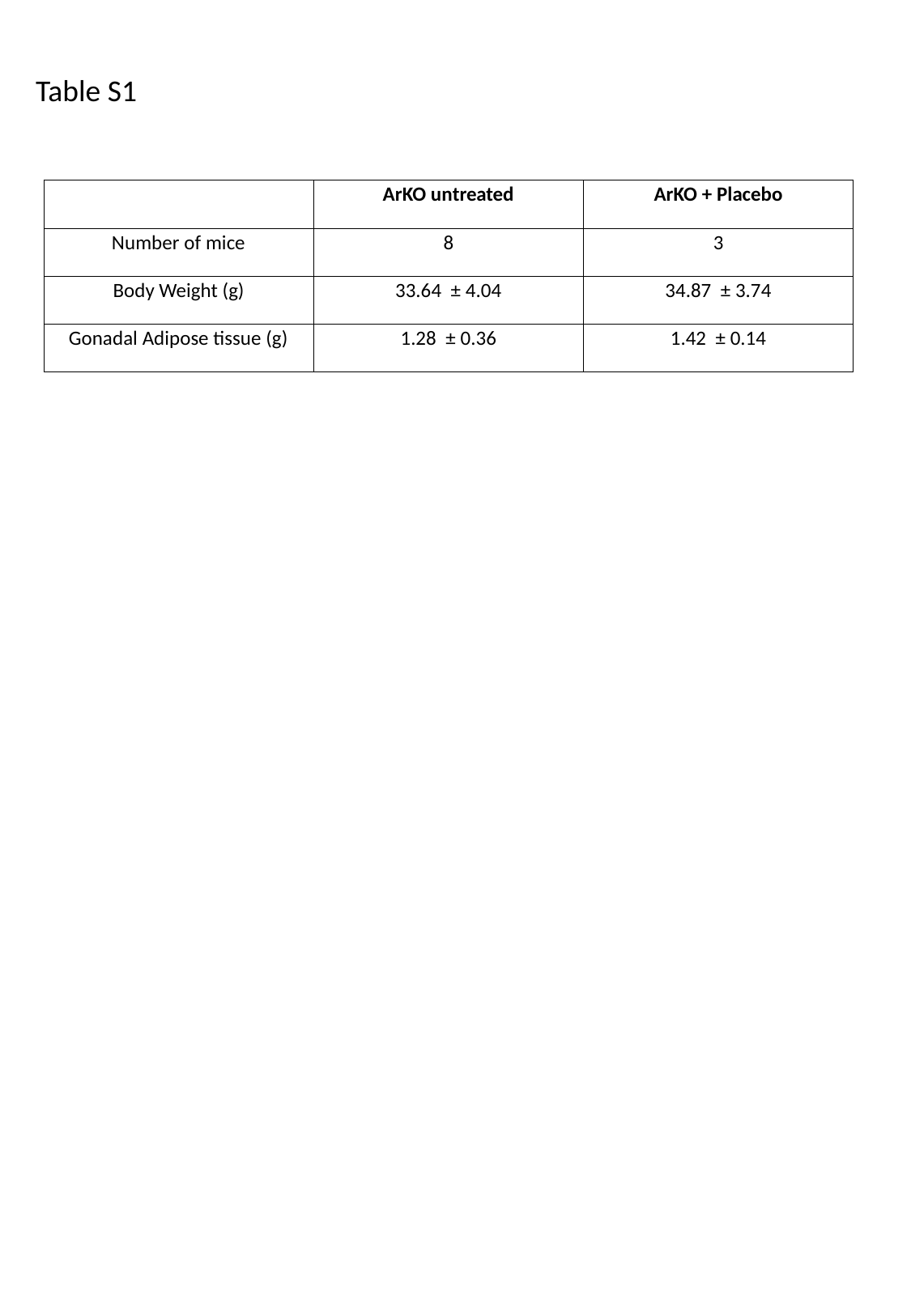

Table S1
| | ArKO untreated | ArKO + Placebo |
| --- | --- | --- |
| Number of mice | 8 | 3 |
| Body Weight (g) | 33.64 ± 4.04 | 34.87 ± 3.74 |
| Gonadal Adipose tissue (g) | 1.28 ± 0.36 | 1.42 ± 0.14 |

Supplement: Table S1 — Body and gonadal tissue weight of untreated versus placebo treated KO mice. Body and gonadal adipose tissue weights in grams (g) of 6 month-old male aromatase knockout untreated (KO) and placebo-treated KO (KOP). Data are expressed as mean ± SD; n = 3–8 per group. (PPT) [file pone.0087230.s003.ppt]
